# A Comparative Study Examining the Management of Bowen's Disease in the United Kingdom and Australia

**DOI:** 10.1155/2015/421460

**Published:** 2015-09-09

**Authors:** G. L. Morley, J. H. Matthews, I. Verpetinske, G. A. Thom

**Affiliations:** ^1^College of Medical and Dental Sciences, University of Birmingham, Edgbaston, Birmingham B15 2TT, UK; ^2^Russells Hall Hospital, Pensnett Road, Dudley, West Midlands DY1 2HQ, UK; ^3^Royal Perth Hospital, 197 Wellington Street, Perth, WA 6000, Australia

## Abstract

*Background and Aim*. The optimum management of Bowen's Disease (BD) is undefined. A review of current practice is required to allow the development of best practice guidelines. *Methods*. All BD cases, diagnosed in one UK centre and one Australian centre over a year (1 July 2012–30 June 2013), were analysed retrospectively. Patients with BD were identified from histopathology reports and their medical records were analysed to collect demographic data, site of lesion, and treatment used. *Results*. The treatment of 155 lesions from the UK centre and 151 lesions from the Australian centre was analysed. At both centres BD was most frequently observed on the face: UK had 70 (45%) lesions and Australia had 83 (55%) lesions (*P* = 0.08). The greatest number of lesions was managed by the plastic surgery department in the UK centre, 72 (46%), and the dermatology department in the Australian centre, 121 (80%). The most common therapy was surgical excision at both centres. *Conclusions*. In both UK and Australia, BD arises on sun-exposed sites and was most commonly treated with surgical excision despite a lack of robust evidence-based guidelines.

## 1. Introduction

Bowen's Disease (BD) is an intraepidermal precancerous lesion. Clinically, it appears as a well-defined erythematous plaque with a scaly or crusted surface, but variants include verrucous and pigmented BD [1]. Histopathology shows full-thickness epidermal dysplasia without dermal invasion [2]. BD is usually indolent and slow-growing; however, there is a small risk of progression to invasive SCC, estimated at 3–5% [3].

BD affects predominantly older individuals, with a peak incidence in the seventh decade [1]. The most commonly affected sites are the face [4], neck, and lower limbs [3, 5].

The strongest risk factor for the development of BD is long-term sun exposure [2]. Further risk factors include fair skin, increased longevity, genetics, and immunosuppression [4, 6].

There are numerous treatment options for BD which can be broadly categorized as surgical or nonsurgical. Surgical treatments include excision, curettage, and Mohs micrographic surgery [4]. Nonsurgical treatments include topical 5-fluorouracil (5-FU), topical Imiquimod, cryotherapy, photodynamic therapy (PDT), ablative laser, and radiotherapy. Observation without active treatment is also a reasonable option in select cases [1, 3].

The British Association of Dermatologists (BAD) in the UK [1] and the Cancer Council in Australia [7] have each produced national guidelines to aid their respective clinicians' therapy choices for the treatment of BD. Both guidelines allow for much clinical interpretation because there is no single modality that can be regarded as optimum for BD management [3]. There are many variables which affect the optimum choice of treatment in an individual patient, including patient age, body site, number of lesions, size of lesions, presence of adnexal extension, failure of previous treatments, cosmesis, cost, local availability, and patient preference [1, 3]. There is a paucity of prospective trials and head-to-head comparisons of the various treatment modalities, and hence literature reviews on this topic [5, 8] have been unable to reach firm conclusions about the comparative effectiveness of treatments.

This study aims to describe and compare current clinical practice for the treatment of BD between one centre in the UK and one centre in Australia.

## 2. Methods

This retrospective comparison study selected patients from centre 1, a public district general hospital in the West Midlands, UK, and centre 2, a tertiary referral public hospital in Perth, Western Australia. All cases of BD of the skin diagnosed in each centre's histopathology department over a 12-month period (1 July 2012 to 30 June 2013) were identified from the histopathology departmental databases. This included both partial biopsy specimens and full excisional specimens, collected by any department within the hospital. The patients' medical records were retrieved, and the following information was collected for each lesion: patient age, gender, site of lesion, dermoscopy use, whether the specimen was a definitive excision versus diagnostic biopsy, treatment modality used, and specialty managing the lesion. Data was recorded in two Microsoft Excel spreadsheets, one for each centre. The results from each centre were compared using frequency tables, and statistical analysis was conducted including Student's *t*-test and the chi-squared test.

Ethical approval was granted for this study by the Audit and Research Department at both centres.

## 3. Results

In the UK centre, there were 193 lesions among 185 patients during the study period. Of those, medical records were available for 149 patients (81%), allowing data collection on 155 lesions (80%). In the Australian centre, there were 200 lesions among 124 patients, and medical records were available for 98 patients (79%), allowing data collection on 151 lesions (76%) (Table 1).

### 3.1. Demographics

In both cohorts, there was a small majority of males: 83 (56%) in the UK and 58 (59%) in Australia (*P* = 0.58). The mean age in the UK was 79 (±9), and the range was 40–97 years, while in Australia the mean age was 76 (±12), and the range was 36–99 years (*P* = 0.03) (Table 1).

### 3.2. Lesion Site

In both the UK and Australia, the most common site for a BD lesion was the face: 70 (45%) lesions and 83 (55%) lesions, respectively (*P* = 0.08). The second most frequent lesion site for both centres was the lower leg: 41 (26%) lesions in the UK cohort and 21 (14%) lesions in the Australian cohort (*P* = 0.006) (Table 2).

### 3.3. Specialty Managing the BD Lesions

The majority of lesions (80%) in the Australian centre were managed by the dermatology department, compared with only 42% of lesions managed by the dermatology department in the UK centre (*P* = 0.001). In the UK centre, the greatest number of lesions (46%) was managed by the plastic surgery department, compared with only 17% of lesions managed by the plastic surgery department in the Australian centre (*P* = 0.001). In the UK centre, a number of BD lesions, 15 (10%), were managed in the community by general practitioners (GP) using the hospital pathology lab for diagnostic purposes. Finally, 1 lesion was managed by the maxillofacial department.

Dermoscopic examination was recorded for 107 (69%) lesions in the UK and 124 (82%) lesions in Australia. For those lesions with no record of dermatoscopic observation in the notes, it was deemed unknown as to whether it was carried out. The treatment choice of lesions did not appear to be associated with dermatoscopy examination which may be due to the majority of lesions being completely excised at biopsy.

### 3.4. Biopsy Types and Treatment Modalities Used

The lesions captured in the data collection included both outright excisions, curetting specimens, and partial (diagnostic) biopsies. The excisional specimens accounted for 103 lesions (66%) in the UK cohort and 102 lesions (68%) in the Australian cohort (Table 2). By definition, surgical excision was the treatment modality used for these lesions, and this was the most common treatment modality used in both centres. The majority of the excisions (70%) were performed by the plastic surgery department in the UK centre and by the dermatology department (75%) in the Australian centre. Curettage was used for 8 lesions (5%) in the UK centre but was not used in any cases in the Australian centre.

Partial (diagnostic) biopsies accounted for 52 specimens (34%) in the UK group and 49 specimens (32%) in the Australian group. The subsequent choices for management of these lesions are shown in Table 2. The commonest nonsurgical modality used for biopsy-proven BD was topical 5-FU in both centres: 20 lesions (48%) in the UK centre and 31 lesions (69%) in the Australian centre (*P* = 0.039). Cryotherapy was the next most frequently used nonsurgical therapy for 14 (33%) lesions in the UK and 7 (16%) lesions in Australia (*P* = 0.047). Imiquimod was less commonly used: 5 lesions (12%) in the UK centre and 3 lesions (7%) in the Australian centre (*P* = 0.36).

### 3.5. Lesion Primaries and Recurrences

In the UK, 13 (8.4%) lesions were recurrences and 142 (91.6%) lesions were primaries. There were 17 lesions in Australia which were recurrences (11.3%) and 134 (88.7%) which were primary presentations. During the year of data collection there was 1 facial lesion recurrence in the UK (0.7%) which was initially treated by dermatological excision. On recurrence this lesion was referred to the plastic surgery department for further excision and grafting. In Australia during the year of data collection 3 lesions recurred (2%), 2 facial lesions and 1 foot lesion. The foot lesion was initially treated with Imiquimod; on recurrence this lesion was then treated by excision in the plastic surgery department. One facial lesion was treated with 5-FU and the other with dermatological excision. On recurrence the latter lesion was referred to plastic surgery for a wider resection and the former was treated by PDT under patient request.

## 4. Discussion

This is the first study to compare the current clinical practice for BD management in the UK and Australia. The study assessed a similar population at each site and found that surgical excision was the most frequent treatment. There was a significant difference between the UK and Australia with regard to which specialist was responsible for the therapy.

### 4.1. Limitations of the Study

This study utilized histopathology reports as a basis for case identification and data collection; therefore our data was only able to capture those cases of BD undergoing histopathologic analysis in each centre. It is recognized that additional cases of BD will have been diagnosed and managed on clinical grounds in both centres during the study period, and these cases will not have been captured by our data collection. Our data included a significant number of excisional procedures performed in hospital-based dermatologic surgery and plastic surgery settings, which may lead to an overestimation of the utilization of surgical excision for BD, given that all excision specimens are sent for histopathology and would therefore be captured in our data. In addition, the hospital-based population in both centres may be skewed towards more complex cases rather than being treated in the community by general practitioners. Nevertheless, our data provides useful information in relation to the incidence and management of BD in both centres.

### 4.2. Incidence of BD

In both centres, the patient populations had a mean age in the eighth decade. This is slightly older than the peak incidence of 60–69 years commonly cited in the literature [1, 4]. The Australian cohort differed from the UK cohort with a wider age range and BD occurring in patients as young as 36 years. This is unsurprising given climatic differences and the higher incidence of skin cancer in Australia generally compared with the UK [9]. In both centres, there was a slight predominance of male patients, which is in contrast to the female predominance commonly cited in the literature [1, 4].

The most common site for BD in both centres was the face, which is consistent with chronic UV exposure at this body site and consistent with the findings of other studies [4, 8, 10]. The lower leg is commonly reported as a frequent site for BD [3], particularly in women [5], but this site was slightly less represented by our data, particularly in the Australian cohort (14% of lesions). However, it is possible that clinically typical BD lesions on the lower leg may be treated on clinical grounds, without a biopsy, and such cases would not have been captured by our data.

### 4.3. Surgical Treatment of BD

In both centres, surgical excision was the most commonly used treatment modality for BD, although we recognize that our study design may have led to an overestimation of the use of surgical excision for BD. Nevertheless, surgical excision is accepted as the gold standard for the treatment of BD, particularly in recurrent or resistant lesions or those with adnexal extension [11]. Interestingly, there was a significant disparity between the two centres with regard to the specialist involved in performing surgery: in Australia the majority of excisions were performed by dermatologists, whilst in the UK the majority of excisions were performed by plastic surgeons. This may reflect differences in local practices and referral patterns. Due to the very high incidence of skin cancer in Australia, it is possible that dermatologists in Australia routinely perform a greater number of surgical procedures for skin cancer compared with their counterparts in the UK.

Curettage was utilized less commonly than might have been expected, particularly in the Australian cohort, in which it was not utilized in any cases during the study period. Mohs micrographic surgery was not utilized in either centre during the study period, which likely reflects lack of local availability in the study centres.

### 4.4. Nonsurgical Treatment of BD

The commonest nonsurgical treatments used in this study were (in descending order) 5-fluorouracil, cryotherapy, Imiquimod, and photodynamic therapy. The patterns of usage of these modalities were similar between the two centres.

5-Fluorouracil is typically applied bd for 3–8 weeks with published clearance rates, after 3 months, ranging from 67% to 83% [12, 13]. A longer duration of treatment and application under occlusion [14] appears to be associated with improved cure rates. Imiquimod has been described in a variety of treatment regimens varying from 3 times per week to daily application, with treatment durations up to 16 weeks. Published cure rates for Imiquimod are in the range of 73%–93% [15, 16]. Studies have identified that one cycle of photodynamic therapy can establish complete clearance for up to 93% of lesions after three months [13, 17, 18]. While there is literature to support the use of each of these modalities in BD, there is a paucity of robust comparative literature to support preference for one modality over another. Other factors which may influence the choice of nonsurgical modality include local availability, cost, and patient adherence to treatment.

### 4.5. Conclusions

Although the limitations of this study are recognized, its results have revealed significant points which enhance our understanding of BD in clinical practice. In both the UK and Australia BD arises in the older population on sun-exposed sites, with particular predilection to the face and lower limbs. Surgical excision was the commonest treatment modality used in this study, although we recognize that the study design may have led to an overestimation of the utilization of surgery for BD overall. A variety of nonsurgical treatments were used, and the lack of strong preference for one modality over another reflects the lack of a clearly defined optimum treatment in the published literature and guidelines. A large prospective randomized study comparing treatment modalities would help to provide greater clarity regarding the optimal management of BD.

## Figures and Tables

**Table 1:** Patient demographics.

	UK	Australia
Number of patients included	149	98
Number of patients not included	36	26
Total patients	185	124
Number of BD lesions	155	151
Number of BD lesions not included	38	49
Total lesions	193	200
Number of males	83	58
Number of females	66	40
Mean age in years (SD)	79 (±9)	76 (±12)
Age range in years	40–97	36–99

**Table 2:** Frequency of treatment modality usage at each lesion site.

Lesion site	Treatment modality used																	
	Surgical excision^*∗*^		Curettage		5-FU		Imiquimod		Cryotherapy		PDT		Radiotherapy		No treatment^*∗∗*^		Total BD lesions	
	UK	Aus.	UK	Aus.	UK	Aus.	UK	Aus.	UK	Aus.	UK	Aus.	UK	Aus.	UK	Aus.	UK	Aus.
Abdomen	1	1	0	0	0	0	0	0	0	0	0	0	0	0	0	0	1	1
Arm	2	1	0	0	1	0	0	0	1	1	0	0	0	0	0	0	4	2
Face	44	53	3	0	9	20	4	2	8	2	2	3	0	1	0	2	70	83
Foot	2	2	0	0	1	1	0	1	0	1	0	0	0	0	0	0	3	5
Forearm and wrist	4	9	0	0	0	4	0	0	1	1	0	0	0	0	0	0	5	14
Hand	10	8	0	0	0	1	0	0	0	0	0	0	0	0	0	1	10	10
Thigh	2	1	1	0	0	1	0	0	0	0	0	0	0	0	0	0	3	2
Lower leg	26	15	3	0	8	3	0	0	3	2	1	0	0	0	0	1	41	21
Neck and chest	9	8	0	0	1	1	1	0	0	0	0	0	0	0	0	0	11	9
Shoulder and back	3	4	1	0	0	0	0	0	1	0	0	0	0	0	2	0	7	4
Total treatment modality	103	102	8	0	20	31	5	3	14	7	3	3	0	1	2	4	155	151

^*∗*^Surgical excision includes all lesions excised by the plastic surgery department, dermatology department, general practitioners, and maxillofacial department.

^*∗∗*^No treatment: observation follow-up/did not attend/deceased.

Aus. = Australia; UK = United Kingdom.
